# Multidrug Resistance Genes Carried by a Novel Transposon Tn*7376* and a Genomic Island Named MMGI-4 in a Pathogenic Morganella morganii Isolate

**DOI:** 10.1128/spectrum.00265-22

**Published:** 2022-05-05

**Authors:** Xing-Wei Luo, Pei-Yi Liu, Qing-Qing Miao, Rong-Jia Han, Hua Wu, Jian-Hua Liu, Dan-Dan He, Gong-Zheng Hu

**Affiliations:** a College of Veterinary Medicine, Henan Agricultural University, Zhengzhou, People’s Republic of China; USDA-ARS

**Keywords:** *Morganella morganii*, multidrug resistance, *dfrA24*, transposon, genomic island

## Abstract

Antimicrobial resistance in Morganella morganii is increasing in recent years, which is mainly introduced via extra genetic and mobile elements. The aim of our study is to analyze the multidrug resistance (MDR) and characterize the mobile genetic elements (MGEs) in M. morganii isolates. Here, we report the characteristic of a pathogenic M. morganii isolate containing multidrug resistance genes that are mainly carried by a novel transposon Tn*7376* and a genomic island. Sequence analysis suggested that the Tn*7376* could be generated through homologous recombination between two different IS*26*-bounded translocatable units (TUs), namely, module A (IS*26*-Hp-IS*26*-*mph*(A)-*mrx*(A)-*mphR*-IS*6100*-*chrA*-*sul1*-*qacEΔ1*) and module B (IS*CR1*-*sul1*-*qacEΔ1*-*cmlA1*-*aadA1*-*aadB*-*intI1*-IS*26*), and the genomic island named MMGI-4 might derive from a partial structure of different original genomic islands that also carried IS*26*-mediated TUs. Notably, a 2,518-bp sequence linked to the module A and B contains a 570-bp *dfrA24* gene. To the best of our knowledge, this is the first report of the novel Tn*7376* possessing a complex class 1 integron that carried an infrequent gene *dfrA24* in M. morganii.

**IMPORTANCE** Mobile genetic elements (MGEs), especially for IS*26*-bounded translocatable units, may act as a reservoir for a variety of antimicrobial resistance genes in clinically important pathogenic bacteria. We expounded this significant genetic characteristic by investigating a representative M. morganii isolate containing multidrug resistance genes, including the infrequent *dfrA24*. Our study suggested that these acquired resistance genes were mainly driven by IS*26*-flanked important MGEs, such as the novel Tn*7376* and the MMGI-4. We demonstrated that IS*26*-related MGEs contributed to the emergence of the extra gene *dfrA24* in M. morganii through some potential genetic events like recombination, transposition, and integration. Therefore, it is of importance to investigate persistently the prevalence these MEGs in the clinical pathogens to provide risk assessment of emergence and development of novel resistance genes.

## OBSERVATION

Morganella morganii, belonging to the tribe *Proteeae* of the *Enterobacteriaceae*, is a facultative anaerobic rod Gram-negative enteric bacterium (1). This bacterium is recognized as an opportunistic pathogen that can cause infections in hospitalized patients due to the presence of its virulence factors, including urease, hemolysins, and lipopolysaccharide (2, 3). In addition, the dissemination of M. morganii may be advanced because of its wide distribution in nature and commendable adaption (4), which poses a serious threat in both humans and animals. In recent years, antimicrobial resistances are mainly induced via extra genetic and mobile elements, which lead to an increasing resistance development of M. morganii isolates (1). It’s reported that infections caused by multidrug-resistant or extensively drug-resistant (XDR) M. morganii often result in clinical treatment failure (5, 6). Here, we recovered a multidrug resistant pathogenic M. morganii isolate carrying a novel composite transposon, designated Tn*7376*, and a IS*26*-containing resistance island, named M. morganii genomic island 4 (MMGI-4).

**FIG 1:**
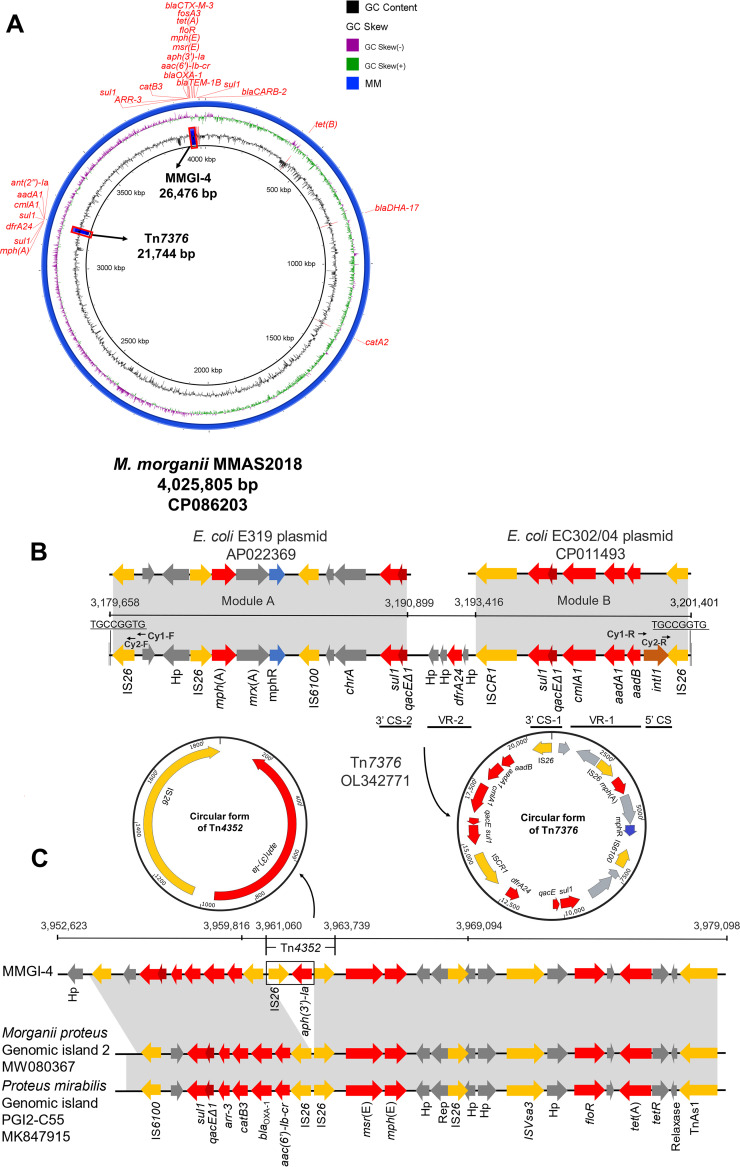
Genomic analysis of M. morganii isolate MMAS2018. (A) Distribution of various antimicrobial resistance genes, the transposon Tn*7376*, and the genomic island MMGI-4 in the MMAS2018. (B) Structural comparison of the Tn*7376* with the homologous regions of the plasmid of E. coli E319 (AP022369) and the plasmid of E. coli EC302/04 (CP011493). TGCCGGTG, indicates an 8 bp direct repeat sequence. Arrows show the direction of each primer and the corresponding positions of the primers along the linear sequence of Tn*7376*. (C) Genetic structure of MMGI-4 in comparison with that of genomic island 2 of *Morganii* proteus (MW080367) and the chromosome of proteus mirabilis (CP043332). Colored arrows represent open reading frames, such as resistance genes in red, mobile elements in yellow, *mphR* (macrolide 2’-phosphotransferase) in blue, *intI1* (class 1 integrase) in orange, and others, including Hp (hypothetical proteins) in gray. 5′ CS, 5′ conserved segment; 3′ CS, 3′ conserved segment; VR, variable region; *qacEΔ1*, quaternary ammonium compound efflux SMR transporter QacE delta 1.

By the reanalysis of antimicrobial resistance in pathogenic bacteria from a swine, we collected a gentamicin- and trimethoprim-resistant isolate from an anal swab sample of a deceased pig that suffered from severe symptoms of diarrhea with visible perianal wound before decease in Henan Province, China, in 2018, which was further identified as M. morganii using matrix-assisted laser desorption/ionization time-of-flight mass spectrometry (MALDI-TOF MS; AXIMA Performance, SHIMADZU, Japan) and then named MMAS2018. A previous study indicated that M. morganii has been historically susceptible to various antimicrobials, including aminoglycosides, carbapenems, quinolones, and trimethoprim (7), so we supposed the isolate MMAS2018 was a peculiar M. morganii strain. Therefore, the isolate was subjected to antimicrobial susceptibility testing (AST) and MICs for various antimicrobial agents, including gentamicin and trimethoprim were determined according to CLSI criteria (8). Resistant breakpoints were interpreted according to previous reports (9). Escherichia coli ATCC 25922 was used as the quality control strain. The whole genome of the isolate was sequenced using Illumina NovaSeq and Oxford Nanopore Technologies (ONT) platforms (Personalbio Technology Co., Shanghai, China). The chromosome sequence was obtained by *de novo* assembly conducted using HGAP v4 and CANU v1.7.1. The resistome was investigated using ResFinder 4.1 (https://cge.cbs.dtu.dk/services/ResFinder/). The chromosome sequence was initially annotated using the RAST server (https://rast.nmpdr.org) and corrected manually using BLAST (https://blast.ncbi.nlm.nih.gov/Blast.cgi). IS elements were identified using ISfinder (https://isfinderbiotoul.fr/). As expected, this isolate showed a multiple resistance pattern by AST, shown in Table 1. The isolate exhibited resistance to all the tested antimicrobials. Furthermore, the isolate contained a full chromosome of 4,025,805 bp, with a G+C content of 51.07%. Whole Genome Sequencing (WGS) analysis showed that MMAS2018 harbored 25 antimicrobial resistance genes, including 4 copies of *sul1* genes (Table 1 and Fig. 1A).

**TABLE 1:** Resistance phenotype and genotype of M. morganii MMAS2018

Antimicrobials	MIC (μg/mL)	Associated resistance gene(s)
Ciprofloxacin	128	*aac(6’)-Ib-cr*
Gentamycin	256	*ant(2’’)-Ia*
Streptomycin	> 256	*aadA1*
Kanamycin	256	*aph(3′)-Ia*
Azithromycin	64	*mph*(A)
Erythromycin	> 256	*msr*(E), *mph*(A), *mph*(E)
Tetracycline	512	*tet*(A), *tet*(B)
Fosfomycin	128	*fosA3*
Sulfamethoxazole	> 512	*sul1*
Trimethoprim	> 512	*dfrA24*
Trimethoprim/sulfamethoxazole	> 64/1216	*sul1*, *dfrA24*
Rifampicin	> 512	*arr-3*
Amoxicillin	> 512	*bla*_TEM-1B_, *bla*_DHA-17_, *bla*_CARB-2_, *bla*_OXA-1_, *bla*_CTX-M-3_
Cefotaxime	> 128	*bla*_CTX-M-3_
Chloramphenicol	> 256	*floR*, *cmlA1*, *catA2*, *catB3*
Florfenicol	> 256	*floR*

Interestingly, 7 out of the 25 resistance genes, including *aadB*, *aadA1*, *cmlA1*, *dfrA24*, *mph*(A) and two copies of *sul1* genes, were carried by a novel IS*26*-flanked composite transposon that was designated Tn*7376*, according to the nomenclature of transposons (https://transposon.lstmed.ac.uk/). Tn*7376* is 21,744 bp in length, corresponding to bases 3,179,658 to 3,201,401 in GenBank accession no. CP086203, which inserted into the chromosomal genome with an 8 bp direct repeat (DR, TGCCGGTG). This transposon mainly consists of two modules, namely, module A and B (Fig. 1B). The module A, IS*26*-Hp-IS*26*-*mph*(A)-*mrx*(A)-*mphR*-IS*6100*-*chrA*-*sul1*-*qacEΔ1*, displayed 100% nucleotide identity to a reference sequence with 100% query cover in an E. coli plasmid (AP022369). Similarly, module B, IS*CR1*-*sul1*-*qacEΔ1*-*cmlA1*-*aadA1*-*aadB*-*intI1*-IS*26*, showed 100% nucleotide identity to the other referenced sequence (CP011493). Although arrangement modes of these resistance genes were common in E. coli, this hybrid transposon Tn*7376*, generated by two different segments of mobile genetic elements (MGEs) from different isolates, made them especial genetic information in chromosome of M. morganii. As previously reported, homologous recombination events are often invoked as the mechanism responsible for the formation of regions containing IS*26*-bounded transposons (10, 11). We predicted that the IS*26*-flanked Tn*7376* was a novel transposon generated through homologous recombination based on these structural features obtained in this study. Importantly, the module A and the module B were adjoined by a 2,518-bp sequence (corresponding to bases 3,190,899 to 3,193,416 in CP086203) carrying an infrequent resistance gene *dfrA24* that encodes dihydrofolate reductase mediating trimethoprim resistance. To the best of our knowledge, only in E. coli strains the *dfrA24* gene was detected (12, 13). By BLAST analysis, we found the 2,518-bp sequence only contained a known open reading frame, namely, a 570-bp *dfrA24* gene. And the gene shows above 99% identity to the corresponding ones of two sequence records in GenBank. Differently, the two referenced *dfrA24* genes are both 558 bp in length and harbored by E. coli strains (NG_047720 and AJ972619). Therefore, this is first description of *dfrA24*, and the gene has begun to spread as a variant in a M. morganii isolate. Notably, *dfrA24*-carrying isolate MMAS2018 showed a high-level resistance to trimethoprim with a MIC of >512 μg/mL and trimethoprim-sulfamethoxazole with a MIC of > 64/1216 μg/mL. The *dfrA24* adjoining module A and module B was located on a novel composite transposon Tn*7376*, which was different from a previous report that showed *dfrA24* was not associated with known mobile elements (12). Interestingly, these resistance genes located on Tn*7376* except *mph*(A) were possessed by a complex class 1 integron consisting of a 5′ conserved segment (5′ CS) (*intI1*), two 3′ CS (*qacEΔ1*-*sul1*), a common region IS*CR1* and two variable regions (named VR-1 and VR-2) containing genes, including *dfrA24* (Fig. 1B). Altogether, the above-mentioned MGEs, including the transposon and the integron undoubtedly exhibited an important biological significance for the dissemination of *dfrA24* in M. morganii. Furthermore, genetic relatedness of MMAS2018 is distantly related to those referenced strains harboring other subtypes of *dfrA* by analyzing the phylogenetic tree of representative M. morganii isolates (Fig. S1 and details shown in supplemental material), which may imply that the *dfrA24*-carrying M. morganii isolate evolved independently.

A translocatable unit (TU), defined as the unit of movement for IS*26*-flanked transposons, of which circular intermediate containing multiple resistance genes is reporting in recent studies (11). To investigate the functional activity, nested PCR was conducted to detect the circular intermediate by amplifying inversely the containing IS*26*-bounded sequence of Tn*7376* (Fig. 1B), using the primers listed in Table S1 (supplemental material): Cy1-F and Cy1-R, Cy2-F and Cy2-R. As a result, a 20,924-bp circular intermediate was obtained by Sanger sequencing and assembly analysis using primer set Cy2-F and Cy2-R (Fig. 1B), suggesting that Tn*7376* could be excised from the chromosomal DNA and form a circular intermediate. Conjugation experiments showed that Tn*7376* in isolate MMAS2018 could not be mobilized to E. coli J53, despite three independent attempts. Taken together, Tn*7376* might act as a reservoir for multidrug resistance genes and potentially contribute to the dissemination of these genes due to the presence of IS elements (9).

In addition to Tn*7376*, we identified a resistance island in isolate MMAS2018 using IslandViewer 4 (14), named MMGI-4, which is 26,476 bp in length (corresponding to bases 3,952,623 to 3,979,098 in CP086203) and carries 10 various antimicrobial resistance genes, including *sul1*, *arr-3*, *catB3*, *bla*_OXA-1_, *aac(6’)-Ib-cr*, *aph(3′)-Ia*, *msr*(E), *mph*(E), *floR* and *tet*(A) (Fig. 1C). Sequence analysis revealed that MMGI-4, except the sequence containing IS*26*-*aph(3′)-Ia* array, was almost identical (>99%) to the sequence from bases 40,353 to 63,615 in *Morganii* proteus genomic island 2 (MW080367) and the sequence from bases 38,886 to 62,730 in Proteus mirabilis genomic island PGI2-C55 (MK847915). It was reported that PGI2-C55, a new genomic island PGI2 variant, of which backbone was identical to that of PGI2. Also, a partial backbone of PGI2 was almost identical (98%) to that of Salmonella genomic island SGI1 and Acinetobacter genomic island AGI1, respectively (15, 16). These results suggested that MMGI-4 might derive from a partial structure of different original genomic islands. Furthermore, the MMGI-4 contained an additional IS*26*-mediated TU (IS*26*-*aph[3′]-Ia*, marked in a rectangular box) that exactly belongs to the known transposon Tn*4352* by combination with the adjacent IS*26* of *aph(3′)-Ia* (17). Consistently, Tn*4352* could be detected to form a circular intermediate by inverse PCR according to the previous report that a circular form containing only an IS*26* and a 1,040-bp segment was generated from this transposon (11). Except the two copies of IS*26* flanking Tn*4352*, the other two IS*26* in a opposite orientation bound a larger composite transposon in size (corresponding to bases 3,959,816 to 3,969,094 in CP086203) that carries additionally *msr*(E) and *mph*(E) genes (Fig. 1C). Based on the presence of these transposons, we supposed that IS*26* elements contributed to the diversity of MDR regions in MMGI-4 through recombination events (18).

In summary, M. morganii, a zoonotic human pathogen, is acquiring important MDR genes that combats treatment of its infections and leads to increasing morbidity and mortality rates. Notably, IS*26* facilitates accumulation of MDR genes and producing genetic events. In this study, we found IS*26* mediated a novel composite transposon Tn*7376* carrying an infrequent resistance gene *dfrA24*. Our study suggests that persistent investigations are needed to assess the prevalence of IS*26*-mediated MDR genes in Gram-negative bacteria, including M. morganii, which is significant for prevention and control of the bacterial infections in public health.

### Data availability.

The complete nucleotide sequences of the chromosome and Tn*7376* in M. morganii isolate MMAS2018 recovered in this study have been deposited in GenBank under accession numbers CP086203 (MMAS2018) and OL342771 (Tn*7376*).
