# Supplementary material for: Multidrug Resistance Genes Carried by a Novel Transposon Tn7376 and a Genomic Island Named MMGI-4 in a Pathogenic Morganella morganii Isolate
Source: Microbiol Spectr. 2022 May 5;10(3):e00265-22. doi: 10.1128/spectrum.00265-22 (PMC9241818; doi:10.1128/spectrum.00265-22)
Supplement: SUPPLEMENTAL FILE 1 — Supplemental material. Download spectrum.00265-22-s001.pdf, PDF file, 0.2 MB [file spectrum.00265-22-s001.pdf]

**Table S1.** Primers used in this study

| Primer name | Sequence (5' ~ 3') | Note |
|---|---|---|
| Cy1-F | GTATCACCGTCCAGTACACG | Detection of Tn*7376* circular form |
| Cy1-R | CAATGTGACCTGCGTTGTGT | |
| Cy2-F | CAGCCTTCTGACTTCTGGTG | |
| Cy2-R | CATTCCTGGCCGTGGTTCTG | |

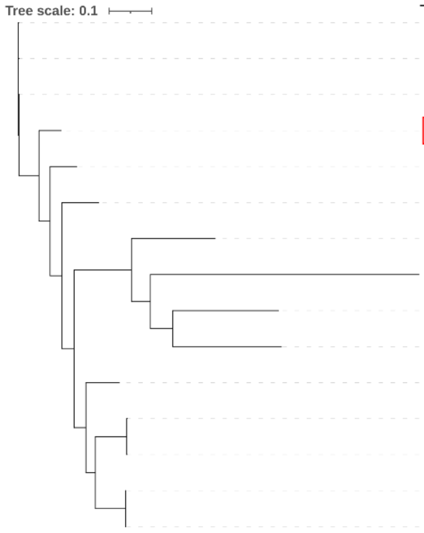


| Accession no. | Strain name | Genotype | Region |
|---|---|---|---|
| CP034944 | ATCC25830 | - | - |
| CP064832 | 12304 | *dfrA12*、*dfrA27* | China |
| CP014026 | FDAARGOS_172 | *dfrA1* | America |
| CP086203 | MMAS2018 | *dfrA24* | China |
| CP059986 | 11759 | *dfrA17* | China |
| CP048275 | N18-00103 | *dfrA12* | Canada |
| CP064830 | 81703 | *dfrA12*、*dfrA27* | China |
| CP028956 | AR_0133 | *dfrA1* | America |
| CP061513 | GDMM86 | *dfrA1*、*dfrA12* | China |
| CP064826 | ZJD581 | *dfrA12* | China |
| CP064829 | 621164 | *dfrA17* | China |
| CP064833 | 715394 | *dfrA19* | China |
| CP070537 | MM48659 | *dfrA1* | Czech Republic |
| CP070524 | MM46903 | *dfrA1* | Czech Republic |
| CP070553 | MM50821 | *dfrA1* | Czech Republic |

**FIG S1** Phylogenetic analysis of MMAS2018 and 13 other *M. morganii* isolates carrying *dfrA* genes. The isolate MMAS2018 and related data were marked in a red rectangular box, and *M. morganii* ATCC25830 was used as a reference.

**Note:** Based on the novel description of *dfrA24* in this study, we employed the whole genome sequences of some representative isolates containing trimethoprim resistance genes deposited in GenBank at NCBI website (https://www.ncbi.nlm.nih.gov/nucleotide/) to construct a phylogenetic tree of *M. morganii* isolates. By thoroughly searching sequences of the complete genome in *M. morganii* in GenBank data, we obtained 14 complete sequences from different isolates that harbors *dfrA* genes. The whole-genome sequence of *M. morganii* ATCC25830 was used as a reference. The core genome of these 15 *M. morganii* strains were subjected to aligning using Parsnp (https://github.com/marbl/parsnp). Creating datasets and constructing the phylogenetic tree were conducted in Interactive Tree of Life (iTOL, https://itol.embl.de) and Adobe Illustrator 2020.

According a previous report, the reported subtypes of *dfrA* genes were mainly *dfrA1* and *dfrA19* in *M. morganii* (1). However, other subtypes including *dfrA12*, *dfrA17*, *dfrA24*, and *dfrA27* were also found in chromosomes of *M. morganii* isolates from different regions (Fig. S1), by our retrieval in GenBank database. As a result, we found the genetic relatedness of MMAS2018 isolate is

distantly related to these referenced strains, which may imply that the *dfrA24*-carrying *M. morganii* isolate evolved independently.

1. Liu H, Zhu J, Hu Q, Rao X. 2016. *Morganella morganii*, a non-negligent opportunistic pathogen. Int J Infect Dis 50:10-17. https://doi.org/10.1016/j.ijid.2016.07.006.
